# Local worlds: Vulnerability and food insecurity in the Eastern Cape province of South Africa

**DOI:** 10.4102/jamba.v12i1.830

**Published:** 2020-07-07

**Authors:** Xolisile G. Ngumbela, Ernest N. Khalema, Thokozani I. Nzimakwe

**Affiliations:** 1 Centre for Transdisciplinary Studies, Faculty of Social Sciences and Humanities, University of Fort Hare, East London, South Africa; 2 School of Built Environment and Development Studies, University of KwaZulu-Natal, Durban, South Africa; 3 School of Management, IT and Governance, College of Law and Management Studies, University of KwaZulu-Natal, Durban, South Africa

**Keywords:** food insecurity, vulnerability factors, poverty, unemployment, interventions

## Abstract

The overwhelming finding is that after more than a decade of democracy, the Eastern Cape (EC) province remains trapped in structural poverty. This shows in all aspects of its demographic, health and socio-economic profiles. Methods, measurements and statistics vary, but from the various studies and data sets one can attest that the majority of the population still lives in poverty. Despite the democratic transformation that began in South Africa in 1994, poverty, unemployment and inequality exist today along with the food insecurity that is symptomatic of them. Food insecurity in South Africa varies across its nine provinces, with the EC province frequently measured as the poorest province in the country. This article examines the extent to which the EC can be defined as vulnerable to food insecurity by using a review of current literature. These vulnerabilities are compounded by the environmental vulnerability factors of climate change and drought, which affect households’ ability to grow food. The elderly and children are affected by life cycle vulnerability factors, with children prone to malnutrition and the elderly unable to work to produce food. Race and gender are associated with vulnerability to food insecurity. Most of the people in the EC who are poor and are African, and a high percentage of women-headed households is poor. The vulnerability factors identified suggest that job creation and agricultural productivity may be useful ways of targeting food insecurity. Interventions need to take local contexts into account and focus on particular communities and their unique needs.

## Introduction

Food security in South Africa remains a challenge, with just 30 000 commercial farmers being responsible for most of the country’s food production (Jarana 2018). More than 200 000 smallholder farmers and an estimated 2 million subsistence farmers have an important role to play in food security and poverty reduction, yet their access to markets, information and finance is limited or non-existent. There is also a lack of available data on smallholder farmers and their supply chains, which is a barrier to informed decision-making by agribusinesses and policy makers. Jarana further argues that the Eastern Cape (EC) province has remained a significantly rural province with a very rich socio-economic and political history from which future generations can draw strength. This province has a crucial role to play in demonstrating how rural people can themselves design their own economic freedom. The people in this province have access to the important natural resources, including land and the human capital. The people need a kick start into the wealth-creating space where rural development finance and economic empowerment are made possible. In South Africa, food security has been a central feature of the present government since 1994, with the right to food enshrined in the country’s constitution of 1996 (Section 27; Section 28 [1c]; Section 35 [2e]). South Africa has made efforts to meet the challenge of food insecurity. However, such efforts are inhibited by the fact that gaining access to food security is influenced by multiple factors and is dynamic in nature. According to development experts and poverty commentators, the major challenge facing food insecurity is that food security interventions lie inadequately on food insecurity assessment and monitoring systems. Addressing the challenges posed by food insecurity requires a thorough understanding of vulnerability and food insecurity conditions at a household level. Being able to identify those who are the most vulnerable and their coping and survival strategies will help government officials to design appropriate relief and development intervention activities. This article provides an overview of the food insecurity of the EC province in South Africa. Using the vulnerability factors identified from a wide range of scholars, this article examines the extent to which the EC can be defined as vulnerable to food insecurity.

## Methods

The researcher adopted a mixed methodology research paradigm in this study for complementary purposes as clearly articulated by Gay and Airasian (2000) and to better capture the different facets of this study according to Sandelowski (2000).

### Study area

This study was conducted in the EC, the most populous and poor province in South Africa. Social and cultural contexts that drive poverty are prevalent in the EC. The province is characterised as a developing province that is entirely dependent on the automotive sector, that is Mercedes Benz South Africa (East London), Volks Wagen and Ford (Port Elizabeth) with two special economic zones (SEZs) (Coega in Port Elizabeth and East London). The province has a good health system, but poor implementation is a major challenge despite the National Health Insurance, which is being implemented by the province for the benefit of its citizens, both rural and urban, who are not covered by the medical aids. The majority of citizens live in abject poverty, and the province is also bedevilled by high unemployment and hunger.

### Data sources

In this study, various data sources were used: the analysis of the community surveys of 2007 and 2016 and censuses of 1996, 2001 and 2011. This study heavily relied on the 2016 community survey as it used household questionnaires that collected information on a wide range of topics, including demographic data. This study also used a number of documents that were produced by various intervention agencies such as donor funding and civil society organisations that were residing in the province as well as various government departments whose mandates are to contribute towards the welfare of citizens. Amongst the documents is also from various academic studies that are conducted on the province as a form of thesis and dissertations by these academic centres that are trying to assist the province to find solution to this catastrophic scourge called poverty that is ravaging the EC province.

## Literature review

### Food insecurity

According to Steven Timm’s writing in the public sector magazine (May 2013), South Africa urgently needs new policy ideas to reverse the alarming increase in poverty amongst its population. New figures reveal an increase of 3 million South Africans living in poverty over the last 5 years. More than half (55%) of the population lives on less than R1138.00 ($107.00) a month, up from 53% in 2011. He further mentioned that in a country with 55 million people, 34 million are going without some of the basic necessities such as housing, transport, food, heating and proper clothing. The escalation in hardship and vulnerability reverses the steady progress made since the dawn of democracy. Seemingly the poorest have been hit the hardest. One in four citizens survives on less than R531.00 a month and is not able to afford enough food to keep themselves healthy. This proportion has risen to one in five in 2011.

Both Timm and Mkhize are in full agreement that the global economic slowdown and depressed commodity prices have obviously contributed to the problem. And as a result these factors have raised unemployment to record high levels. Domestic political uncertainties, regulatory conflicts and weaknesses in public administration have also dented business confidence and undermined private sector investment. According to the two authors as quoted by the *Conversation Magazine* and supported by the National Minister of Department of Rural Development and Land Reform (South Africa [SA]), Minister Gugile Nkwinti (National Minister of Agriculture and Land Affairs), new ideas are needed to combat poverty and inequality in South Africa. A good place to start is the concept of inclusive growth. This is partly about ensuring that more people play an active part in economic, social and political processes. It gives people agency and dignity and helps to hold institutions to account.

It is alleged that since the 1990s, the present government’s approach to poverty has focused on fiscal redistribution. The more the people earn, tax rates increase and the revenue raised has been used to support poorer groups. Social assistance has taken the form of free housing, free education, healthcare, sanitation, electricity and social grants. According to some economists and Seepe (SABC [South African Broadcasting Corporation] News August 2017), spending on this ‘social wage’ has more than doubled in real terms (taking inflation into account) over the past decade. It now amounts to 60% of total public spending. South Africa is reputed to have one of the most redistributive fiscal policies (tax and spending) in the world.

Yet, with a sluggish economy, high government debt and new demands for bailouts from state-owned enterprises and universities, the ability to pay for this welfare system is severely strained. It will be extremely difficult to raise the value of social support or extend it to more people in the period ahead. Since South Africa progressed from apartheid to democracy in 1994, there has been an understandable focus on distributional matters. These efforts have taken priority over action to promote broad-based growth, integrated development and overall prosperity. Too little attention has gone into expanding production, employment creation (outside the public sector) and enlarging the resource base. Amongst households, too, there has been excessive emphasis on spending, rather than on saving and investing in education, skills and household assets. As a result, the economy and society are poorly equipped to cope with shocks.

In an effort to try and help the situation, the national department of Rural Development and Land Reform (RSA) describes a situation that shows us that inclusive growth is often touted as a way forward. It is an umbrella concept to bring people with different interests to work together and build a new consensus around a shared agenda for the country’s development. But what does it mean in practice? In a sense the purported central element is believed to be creating new and different kinds of economic opportunities and linking them explicitly to social needs. Major investments in technology, infrastructure and other facilities should lower barriers to inclusion and give more people on low incomes a chance to work or develop skills to progress.

So far it is worth mentioning that the recent discussions on both poverty and food insecurity have been limited to national stakeholders. Equivalent local initiatives have been few and far between. Yet a strong and prosperous society cannot be engineered from above. South Africa needs to build competent institutions at local level too, if we are to win the scourge facing us as both the province and the municipalities. Common platforms for collaboration are required to harness the energies and share the know-how of people in the cities, towns and villages where they live, work and invest.

A place-based approach means tailoring inclusionary economic policies to local realities and opportunities. Local investments in housing, transport, business property and skills will have a greater impact when they are carefully linked and coordinated. Current ambivalent attitudes towards informality are not helping anyone and instead seems to be further distressing the already vulnerable communities. The informal economy could be a nursery for people to learn valuable skills, a stepping stone to formal jobs, and a seedbed for larger, more competitive businesses. Many unemployed and destitute adults cannot receive the grants available to pensioners, children and the disabled. Yet various forms of bureaucratic red tape obstruct their initiatives to start informal enterprises and ‘create their own jobs’. In a spirit of fairly dealing with vulnerabilities and neglect of our immediate communities, it is suggested that informal settlements are a route for people to escape rural poverty and gain access to urban job markets. Yet they are neglected and hazardous places threatened by government evictions. Affordable urban housing could instead be a driver of inclusive growth and spatial transformation.

In an economic slump, with tight public finances as argued by Sipho Seepe (SABC News August 2017) and agreed upon by the *Conversation Magazine*, the government needs new and different ways of responding to local realities. It should orchestrate efforts throughout society which actively supports people’s struggles to get ahead, not just enable them get by.

### Vulnerability

The Food and Agriculture Organization of United Nations defines ‘vulnerability’ as the presence of factors that place people at risk of becoming food insecure or malnourished, including those factors that affect their general ability to cope under any stressful situations they find themselves in. Reliable food is closely linked to notions of sustainability and vulnerability. Vulnerability results when a household has to sacrifice the long-term ability of its members to acquire sufficient food to meet current, short-term needs. Food security incorporates the notion that a household does not have to sacrifice long-term ability to be food secure for short-term needs (Wren & Benson 2004:8).

Both food security and poverty are used to describe people’s welfare state at the present time. Vulnerability complements this static picture with a dynamic forward-looking perspective that is used to predict how the welfare of individuals and households may change in future as a consequence of not being able to face adverse events that may happen to them. Simon (2012) indicates that vulnerability can be expanded to capture a more complex relationship between risks and ability to cope (actions taken before, during and after shocks) that affects food security.

The United Nations Development Programme’s Human Development Report (2014:2) reveals that people over the world are vulnerable, to some degree, to shocks that include natural disasters, financial crises and armed conflicts as well as to long-term social, economic and environmental changes. Yet some people are much more vulnerable than others. In many cases, it is generally believed that discriminatory social norms and institutional shortcomings exacerbate vulnerability, leaving certain groups without the household, community and state support needed to boost their coping capacities. According to the Human Development Report (2014), those living in extreme poverty and deprivation are amongst the most vulnerable. Despite the widely reported progress in poverty reduction in some countries, the report reveals that more than 2.2 billion people are either near or living in multi-dimensional poverty. This means that more than 15% of the world’s people remain vulnerable to multi-dimensional poverty. Food insecurity is inherently associated with poverty and vulnerable groups in communities. Hence, poverty is used as one of the indicators in describing food insecurity (Agricultural Policy Unit 1997).

### Vulnerability factors

Anderson et al. (2011:597) and Gbetibouo et al. (2010) argue that vulnerability relates to the potential and future jeopardy with the implications or likelihood that some kind of crisis may occur that will damage one’s health, life or the property and resources on which health and life depend. People with limited core capabilities and restricted choices are prevented from coping with the above-mentioned threats. Chagutah (2013) affirms that a gamut of social, economic, political and environmental aspects makes up the matrix of factors that mediate vulnerability. This article looks at the different vulnerability factors and assesses the extent to which the EC is subject to each of them.

### Social vulnerability

Social vulnerability factors are mostly linked to the level of well-being of individuals, households or communities. Social vulnerability considers basic needs such as the level of education and literacy, peace and security, access to basic human rights, systems of governance, social equity, positive traditional values, knowledge structures, customs and ideological belief systems and an overall organisational system (Philo 2005:442).

#### Insufficient infrastructure associated with rural areas

The rural nature of some areas in the EC region makes them vulnerable to having insufficient infrastructure that would lead to an overall organisational system. Statistics show that in South Africa, 72% of people live in rural areas, and that 70% of those who live in such rural areas are poor. This status of being poor is ‘exacerbated by the disproportionate lack of access to services’. In addition, the country is characterised by an urban–rural population split of 51% – 41% (Agricultural Policy Unit 1997). The majority of citizens in the EC province live in rural areas.

In most cases, the rural areas in South Africa are dominated by underdevelopment. A primary problem for the EC is that infrastructure is underdeveloped, mostly as a consequence of being poorly maintained. The consequence is the reduction of the value of infrastructure assets (EC Vision 2030 2014:80). This happens despite the fact that the national and provincial governments have plans in place for developing infrastructure in different parts of the country. For instance, according to the Department of Social Development (DSD 2015) the Strategic Plan (National Department of Public Works 2015), R6 billion was planned to be spent on goods, services and infrastructure development by 2016. The value of such services is that they would assist in making improvements to the livelihood of the citizens of the country and therefore result in economic stimulation. However, in most cases the implementation of such plans does not take place as intended, as a consequence of the challenges that arise in rural areas. For example, in areas like the Amathole region in the EC, rural villages are located in areas that have a complicated topography that results in high costs of delivering bulk infrastructure such as water and electricity. Consequently, such areas are characterised by infrastructure backlogs (EC Vision 2030 2014:80).

### Low education levels

The EC has four universities – Walter Sisulu University, the University of Fort Hare, Rhodes University and Nelson Mandela Metropolitan University – and several colleges (Amathole District Municipality [ADM] Vision 2030 2015). Despite the existence of these universities and colleges, most people in the EC province do not have post-matric qualifications. In the absence of this type of qualification, chances of becoming competitive in the job market deteriorate. This situation also contributes in the deterioration of opportunities for self-employment.

### Economic vulnerability

The economic status of most nations, communities, households and individuals greatly influences their level of vulnerability. This status relates proportionately to higher losses in case of a disaster and lower capacity to recover (Anderson et al. 2011:596). The poor are always seen as more vulnerable than the economically better off sections of the society. Wisner et al. (2004:55) state that the economic factors of vulnerability include levels of reserves, debt and degree of access to credit and loans as well as insurance. Wisner et al. (2004) find that the lack of access to basic services such as water forces people to use unsafe sources for cooking and drinking, placing them at risk of epidemics and diseases. The absence of electricity or other sources of power forces people to cut down trees for firewood, which in turn leads to environmental degradation (Barbat et al. 2010:553).

The EC province is still trapped in structural poverty. It is estimated that between 20% and 60% of the province’s population lives in poverty. The highest level of poverty is shown in Alfred Nzo at 57.7%, followed by OR Tambo at 53.9% and, lastly, Joe Gqabi at 49.8%. The area with the lowest poverty rate in the province is the Nelson Mandela Bay Metropolitan Municipality at 28.3% (Eastern Cape Planning Commission 2014). On a positive note, statistics reveal a decline in the level of poverty in the EC districts, between 7% and 24% over the years 2000–2012. This reduction in poverty rates is attributed to the provision of social grants by the South African government (Eastern Cape Planning Commission 2014). The provision of grants does not seem to be a sustainable measure for combatting food insecurity, however, as it creates dependency rather than promoting self-sufficiency.

The problem of low income affects certain districts of the EC. According to the Eastern Cape Planning Commission (2014:27), there is a spatial disparity in terms of income amongst the EC district municipalities, with areas that have a low-income level including the OR Tambo, Amathole and Chris Hani district municipalities.

Low income is associated with unemployment. In Alice, which is in the Raymond Mhlaba Local Municipality in the EC, for example, a 2014 survey on the relationship between employment and food security found that 21% of the unemployed were severely food insecure (Dodd & Nyabvudzi 2014), which was attributed to high levels of unemployment in the area, which resulted in low monthly household incomes. In fact, 62% of the surveyed households reported earning less than R1500 per month. Such a low income tends to influence household food security negatively. In addition, according to Lahiff (2014), opportunities for migrant labour to the mining and industrial sector, on which the EC has long depended, have fallen dramatically in recent years, and many local sources of employment, notably in the public sector, are also shedding jobs. Declining opportunities for formal employment have forced many households to turn to informal activities to obtain a livelihood, including an increased dependency on traditional land-based activities.

The dominance of high unemployment levels, low income and poverty in the majority of the population in certain municipalities has a negative effect on the ability of these municipalities to collect revenue from their citizens. Consequently, revenue collection in these municipalities is low. Ultimately the municipalities become dependent on the national government for grants, as they are not able to build up their own revenues. The challenges of poverty and food insecurity increase in this situation because municipalities are unable to provide effective service delivery to their communities.

### Socio-economic vulnerability

Inadequate access to critical and basic socio-economic infrastructure such as communication networks, utilities and supplies and transportation facilities increases people’s exposure to risk. Economic Capacity Building (Peters 2006) states that lack of awareness of and access to information can increase levels of vulnerability. The Census 2011 index of socio-economic underdevelopment, which is based on indicators for education, income and unemployment, indicates that most of the former Bantustan areas of the EC have higher scores, although levels of development are higher in the western, central and urban parts of the province. This is a clear indication that the structural legacy of the homeland system remains, and that areas such as the OR Tambo and Alfred Nzo districts should remain targets for social and economic interventions. Other examples of areas with low levels of socio-economic development are Mthatha, Queenstown, Butterworth and Fort Beaufort (EC Planning Commission 2014:27; EC Vision 2030 2014:82). For all these regions, socio-economic measures to create jobs, promote self-employment and achieve economic inclusion are paramount for any long-term solution to the problem of widespread poverty.

### Environmental vulnerability

The ecological factors that influence many disasters are either caused or aggravated by environmental degradation. Drought conditions are a natural phenomenon but may be exacerbated by poor cropping patterns, overgrazing, stripping of top soil, poor conservation methods and depletion of both surfaces and subsurface water supplies and unchecked urbanisation (Van den Eeckhaut et al. 2010:348; Nathan 2008:340). For Wisner et al. (2004:56), environmental vulnerability includes the extent of natural resource depletion, state of resource degradation, loss of resilience of the ecological system, loss of biodiversity and exposure to toxic and hazardous pollutants. As mentioned above, deficiencies in service provision such as the absence of electricity or other sources of power create environmental degradation when people cut down trees for firewood, which in turn leads to an increased exposure to flooding and other hazards (Barbat et al. 2010:553).

The world is currently facing huge challenges associated with a changing climate and rapidly growing exposure to disaster risks. For developing countries, which are less able to cope with the impact and more likely to be affected, the challenges are particularly severe. Climatic patterns characterised by cyclic drought, floods and cyclones have become more frequent in Southern Africa. As agriculture is a key driver in the economic growth of countries in the region, the effect on agriculture-based livelihoods is likely to be severe.

### Physical vulnerability

Physical vulnerability factors encompass the aspects of location and susceptibility of the built environment. Kynia et al. (2008:4) describe physical vulnerability as the susceptibility of individuals, households and communities to the physical environment in which they find themselves. Kynia et al. (2008:4) reveal that this type of vulnerability relates to issues such as access to suitable land, land use, land planning, housing design, building standards, materials used for building houses and emergency services. According to Wisner et al. (2004:56) and Mc Entire, Gilmore Crocker and Peters (2010:58), physical vulnerability is increased by remotely located settlements and lack of access to service infrastructure and information.

#### Land tenure

Land tenure is an important variable that affects vulnerability. Chagutah (2013) and Reale and Handmer (2011) claim that vulnerability can occur either where land tenure is perceived to be insecure or where insecure tenure results in the loss of land, especially when alternative livelihood and housing options are limited. A study commissioned by the Economic Commission for Africa (Economic Commission for Africa [ECA] 2003) confirms that land tenure insecurity is still widespread in Southern Africa and manifests itself in a number of ways, for example in the insecurity of farm workers and farm labour tenants in South Africa.

Chagutah (2013) distinguishes two forms of land tenure systems in Southern Africa: customary and statutory tenure systems. The customary land tenure system is governed by unwritten traditional rules and administered by traditional leaders. Households have strong exclusive residential rights, seasonally exclusive rights to arable land and shared rights to grazing land and natural resources. Land is not alienable from the community trust. In contrast, the statutory land tenure system, according to Chagutah (2013), is governed by modern law, is supported by documentary evidence such as a title deed or lease certificate and is administered by the government. Land ownership under the statutory tenure system is often built on freehold or leasehold entitlements to the land and offers exclusive rights to the owner, which guarantee land tenure security. Land rights in freehold include the ability to sell the land, rent it to others or use it as collateral for a mortgage.

Historically the indigenous people of the EC area had a direct relationship with land as their main means of production that supported their agrarian-based livelihoods and stock farming that was regarded as the main means of wealth and well-being. However, according to Mbongwa et al. (1996) and Bundy (1979), the arrival of the white colonial, settler farmers brought with it a total destruction of the social construct and food stability within the area.

#### Agriculture as a livelihood

Although, as mentioned above, 41% of South Africa’s population is rural, agriculture makes a low contribution to the country’s gross domestic product (GDP). According to Statistics South Africa (Stats SA 2011), the percentage contribution to annual 2011 GDP by agriculture, forestry, hunting and fishing was 2.5%. Although this suggests that agriculture and rural livelihoods are relatively unimportant in the food insecurity equation, the national accounts do not convey a clear picture of the importance of land access on the ground (Hart 2009). Jacobs (2008) claims that close to 4.5 million black South African citizens participate in agriculture, with more than five times as many people employed as wage earners in the large-scale commercial farming sector.

That food security is not only an agricultural function is further upheld by Scanlan (2003:89), who maintains that inequality is what leads to food insecurity. Food security is not the consequence of agriculture alone but is a multi-dimensional phenomenon that requires a multi-dimensional approach such as that outlined in the Integrated Food Security Strategy (IFSS) of South Africa. However, the failure of the successful implementation of the IFSS for South Africa supports the narrative that the Department of Agriculture is in favour of commercial agriculture at the national level as its priority agricultural development initiative. This weakens poor people’s ability to acquire food, making them more vulnerable to food insecurity.

### Life cycle vulnerability

The term ‘life cycle vulnerability’ refers to threats that individuals face in different stages of their life, from infancy through youth, adulthood up to old age. In focussing on life cycle vulnerability and the formation of life capabilities, the Human Development Report (2014) draws attention to sensitive phases when a person may be particularly susceptible. Inadequate attention during such periods can limit capabilities and heighten vulnerability.

In South Africa, the age group of people in households or in communities can make them vulnerable to food insecurity. According to the Agricultural Policy Unit (1997), this group includes people who are over 60 or under 18 years of age. For instance, statistics indicate that three in every five children live in households that are considered poor. As an example, the Northern Cape province has 83% of children who live in households that are poor (Agricultural Policy Unit 1997). In South Africa, in 1997 there were approximately 1.5 million malnourished children under the age of 6 years (Agricultural Policy Unit 1997).

### Structural vulnerability

This form of vulnerability, as explained in the Human Development Report (2004:55), is embedded in social contexts. Structural vulnerability focusses on individual and group characteristics, including group identity, that are associated with a higher vulnerability to adverse circumstances. The reduced ability to bounce back can be traced to inadequate investments in building capabilities throughout the entire life cycle, disability, geographical remoteness or other isolation, or to societal barriers that prevent people from realising their potential.

According to the Human Development Report (2004), structural factors can subject people or groups to multiple disadvantages. Group-based discrimination and exclusion exist across multiple dimensions, such as political participation, healthcare, personal security and education, and generate chronic and overlapping vulnerabilities for minorities and other excluded groups by limiting their capabilities and their potential role in society.

In South Africa, the challenge of vulnerability to food insecurity as a consequence of poverty is racially based. Specifically, it seems to be confined to households of Africans. This is because Africans are frequently poor, at 66%, in comparison with less than 2% of households of white people (Agricultural Policy Unit 1997).

The problem of food insecurity in South Africa and in the province has gender dimensions. In most cases women are more vulnerable to food insecurity than men. For example, about 48% of women in comparison with 43% of men live in conditions of poverty in both rural and urban areas. Simultaneously, approximately 67% of poor female-headed households are located in rural areas (Agricultural Policy Unit 1997).

### Ethical consideration

The permission to collect data was obtained from the University of Fort Hare’s Humanities and Social Sciences Research Committee (Ethical Clearance Number: HSS/1462/017D) and the provincial departments agreed on verbal consent as the article will be based on their reports.

## Discussion and findings

The decline in living standards that has been reported by the EC Department of Social Development (DSD 2018) gives a grim picture despite the extension of social grants to 2 million more recipients in the past 5 years, bringing the total number of social grant beneficiaries to 17 million. According to DSD, it is nearly one in three people. Over the previous decade and a half, extending social grants proved to be an effective way of alleviating hardship. Something has changed. The global economic slowdown and depressed commodity prices have obviously contributed to the problem. These factors have raised unemployment to record high levels. Domestic political uncertainties, regulatory conflicts and weaknesses in public administration have also dented business confidence and undermined private sector investment. New ideas are needed to combat poverty and inequality in South Africa. A good place to start is the concept of inclusive growth. This is partly about ensuring that more people play an active part in economic, social and political processes. It gives people agency and dignity and helps to hold institutions to account. Also worth noting regarding the food insecurity challenge is that since the 1990s, the government’s approach to poverty has focused on fiscal redistribution. The more the people earn, tax rates increase and the revenue raised has been used to support poorer groups. Social assistance has taken the form of free housing, free education, healthcare, sanitation, electricity and social grants.

However, according to National Treasury (2017), spending on social wage alone has more than doubled in real terms if taking inflation into account over the past decade. National Treasury further concludes that this amounts to 60% of total public spending. South Africa is reputed to have one of the most redistributive fiscal policies (tax and spending) in the world. Yet, with a sluggish economy, high government debt and new demands for bailouts from state-owned enterprises and universities, the ability to pay for this welfare system is severely strained. It will be extremely difficult to raise the value of social support or extend it to more people in the period ahead. Since South Africa progressed from apartheid to democracy in 1994, there has been an understandable focus on distributional matters. These efforts have taken priority over action to promote broad-based growth, integrated development and overall prosperity. However, it is agreed then that too little attention has gone into expanding production, employment creation (outside the public sector) and enlarging the resource base. Amongst households, too, there has been excessive emphasis on spending, rather than on saving and investing in education, skills and household assets. As a result, the economy and society are poorly equipped to cope with shocks.

South Africa urgently needs new policy ideas to reverse the alarming increase in poverty amongst its population. New figures reveal an increase of 3 million South Africans living in poverty over the last 5 years. More than half (55%) of the population lives on less than R1138.00 ($107.00) a month, up from 53% in 2011. In a country with 55 million people, 34 million are going without some of the basic necessities such as housing, transport, food, heating and proper clothing. The escalation in hardship and vulnerability reverses the steady progress made since the 1990s. The poorest have been hit the hardest. One in four citizens survives on less than R531.00 a month and is not able to afford enough food to keep healthy. This proportion has risen from one in five in 2011. The consequences are plain to see. They include more street begging, homelessness, loan sharks, social discontent, substance misuse and violent crimes in many communities. Income is not the only measure of poverty. Progress on education, health and basic living conditions also seems to have stalled.

Nkwinti (2017), as quoted by the South African-based public sector magazine, remarked that South Africa is a food insecure country where 6% of our citizens face daily hunger and about 30% (almost one-third) have gone hungry in the last 30 days. Yet statistics show only 17.4% drop in agricultural output and only 18.3% of South Africans are involved in any kind of agriculture since the government’s introduction of One Household, One Hectare programme to strengthen and bolster the Brazilian programme. However, the EC Department of Rural Development and Agrarian Reform (DRDAR) is steadfastly promoting agriculture as a game changer for both poverty and food insecurity despite the nationally reported drop in agricultural output and agricultural involvement by the citizens especially the youth and the most middle-income group earners. The EC province is taking a new trajectory to embrace commercialisation of agriculture in rural communities by targeting smallholder and communal farmers to successfully derive optimal economic value out of their agricultural activity through customised partnerships with organised commercial partners. This approach will provide investment, technology capabilities, training and mentoring and capital to promote transformation in the sector. The recent trend of declining national and provincial economies relative to the growth rates required to service the development agenda to address the scourge of socio-economic inequality, poverty and underdevelopment in the country is an increasing threat to stability in the country. A brief overview of the economic status and trends in associated factors that have direct bearing in the socio-economic trends is set out below.

Given all the above arguments, South Africa’s performance is at just above average when it comes to world food security ranks by the London-based newspaper called *The Economist*. According to National Development Plan (NDP) (2013) as quoted by public sector manager magazine in 2012 the Global Food Security Index published by the Intelligence Unit of the London-based *The Economist* newspaper ranked South Africa 40th out of 105 countries when it comes to food security with the US ranked at first spot. Timm strongly upholds that South Africa has been having sufficient food in most years and supported by the NDP which enunciates we have been producing enough maize for all but three of the past 50 years with exceptions being the droughts of 1984, 1992 and 2007. The NDP (2013) also suggests that agricultural sector should adopt innovative measures such as buying from more small-scale farmers to create local buffer stocks and community-owned emergency services. While the NDP (2013) is suggesting to the agriculture sector to adopt innovative measures, the Department of Agriculture, Forestry and Fisheries is currently looking at establishing a significant number of smallholder producers by helping land reform beneficiaries through its comprehensive Agricultural Support Programme. The DRDAR further proposes that along with the other stakeholders departments like Trade and Industry (DTI), Social Development (DSD) and Rural Development should be running public private programmes based on the Brazilian model of Zero Hunger programme. The Brazilian programme of Zero Hunger, which is regarded as highly successful, was founded on the basis of helping households and farmers improve food production. Mkhize, who is a food security and agrarian reform expert employed by DRDAR, revealed that South Africa has emulated the Brazilian model with South African programme targeting about 23 highly deprived district municipalities across the country. He (Mkhize) further estimated that more than 235 000 hectares of land have been under production which yielded mostly maize but also dry beans and potatoes using state support as emulated from the Brazilian experience of Zero Hunger programme. According to his claims, more than 15 000 smallholder and subsistence farmers were receiving support in the form of tractors, fertilisers and agricultural implements as well as training and market linkage support. Certainly if the Brazilian experience is regarded as one of the best practices that are available out there to curb the underlying dynamics of both food insecurity and vulnerability in Amathole and elsewhere for that matter, government departments and public bodies need to bolster planning and research efforts and stay on top of monitoring and administering the precious resources of food and water self-consciously without any fail so that we do not fail the future generations. And clearly and most importantly it is crystal clear that South Africans can no longer continue to take the availability of food and other resources for granted anymore if we are serious about the country’s welfare and future at heart.

## Malnutrition situation in the province

According to some statistics from the District Health Information Systems (DHIS 2017) released by the EC Department of Health and also confirmed by a local newspaper, seven people died of herbal intoxication because they were trying to eat some wild plants (inquest to be conducted to address food insecurity in the province). Some district municipalities had to hastily establish a Monitoring and Response Unit (MRU), which is a multi-disciplinary forum to assist and contain the poverty situation that has resulted into these acute malnutrition deaths because of the seven reported cases of herbal intoxication. This forum is to meet monthly to discuss strategies to be implemented as there was no freely available strategy to deal with the herbal intoxication cases presented by desperate patients in their facilities. As generally believed and hoped for the forum that comprises South African Social Security Agency (SASSA), DSD will assist in the offering of food parcels, social grants (child grant, foster care) as an immediate shock absorber although the DSD is looking at the long-term sustainable solution and partners. Department of Rural Development and Agrarian Reform’s conspicuous absence from assisting households with food gardens, despite this province’s tagline of making agriculture a game changer, leaves a lot to be desired. However, the situation has summarily encouraged the Department of Health to realise the importance of community health workers who are now charged to do Mid Upper Arm Circumference (MUAC) measurements to identify the children early to prevent them from experiencing severe acute malnutrition (SAM). Amongst the decisive solutions is the greater involvement of the nutritional managers who will be attending the war room meetings where they will be presenting the nutritional status and plans developed to further guide the wards towards the realisation of food security through food gardens (Table 1).

**TABLE 1:** Acute malnutrition deaths in the Eastern Cape hospitals from 2013 to 2017 (reported cases).

District	Year							
	2013/2014		2014/2015		2015/2016		2016/2017	
	Adm.	Deaths	Adm.	Deaths	Adm.	Deaths	Adm.	Deaths
Buffalo City Metro	201	27	252	24	231	27	181	14
Nelson Mandela Metro	173	17	198	25	189	16	139	12
Amathole	311	27	248	35	222	31	190	20
Chris Hani	455	44	422	42	378	31	399	23
OR Tambo	703	154	1029	119	1160	128	837	99
Alfred Nzo	296	55	381	69	310	37	250	35
Joe Gqabi	175	28	159	18	181	8	127	18
Sarah Baartman	211	4	178	7	148	6	98	5

**Total**	**2534**	**356**	**2867**	**339**	**2819**	**284**	**2221**	**226**

*Source*: District Health Information Systems (DHIS), 2017, *Eastern Cape District Health Information System*, Bhisho.

Adm., admissions.

The strengthening of social mobilisation will be undertaken through road shows for educating mothers and caregivers on infant feeding, which will be conducted during the first and second days of each month to curb child and infant mortality. Districts are also charged with the task of organising a workshop for traditional health practitioners to educate them on signs and symptoms of undernutrition and the 16 key family practices. The inclusive approach of the province that is so reactionary and sloppy also saw the late inclusion of the traditional leaders through the involvement of *Imbumba YamaKhosikazi AkoMkhulu* (IYA). This opinion piece has also noted with concern that community nutrition development centres (CNDCs) are not covering the most needy areas and are not clearly monitored by the DSD, hence, the sporadic episodes of deaths from both SAM and herbal intoxication by communities that are under food insecurity pressure and vulnerability of not coping with hunger and despair of not knowing where their next meal is going to come from.

## Conclusion

The analysis presented in this article shows the EC to be vulnerable on a number of fronts. As Lahiff (2014) points out, the transition to democracy in South Africa, which began in 1994, has only begun to reverse the legacy of the past. Poverty and vulnerability associated with land tenure issues, unemployment, lack of basic services and, above all, poverty remains central to the lives of the majority of the population of the EC. In addition, the ‘deep rural’ areas of the former apartheid ‘Bantustans’ of the Ciskei and, most especially, the former Transkei have presented enormous challenges to the reform policies introduced by the state since 1994, including the provision of the social infrastructure services of electricity and water.

The areas of vulnerability identified indicate that food insecurity at household and individual levels in rural areas is best addressed by job creation and agricultural productivity. Other strategies include rural infrastructure development. Vulnerable groups, particularly the elderly and women, should have access to nutritional services through the health system and social relief-of-distress programmes. According to Ngumbela (2015), interventions must be appropriate to local contexts, and the existing strategies for supporting food security must be relevant to the targeted community and unique in their objectives and design. The literature consulted reveals that food security is a complex societal issue that requires urgent practical solutions and appropriate, targeted policies and multi-pronged interventions. According to the University of KwaZulu-Natal’s African Centre for Food Security (2017), food security requires transdisciplinary education and research, which includes searching for practical solutions to food security, development of practical, reliable measures of household food security, evaluation of interventions and influencing policy development and analysis in Africa.

If the above is taken seriously and the province’s policy makers are to make a serious dent in food insecurity, new ways of research and advocacy regarding food security and related areas require greater integration of perspectives in the identification, formulation and resolutions of shared problems so that more efficient and comprehensive assessment and interventions can be provided. The insights gained here indicate the value of further research by using this approach.
